# Supplementary material for: Studying biomolecular folding and binding using temperature-jump mass spectrometry
Source: Nat Commun. 2020 Jan 28;11:566. doi: 10.1038/s41467-019-14179-x (PMC6987177; doi:10.1038/s41467-019-14179-x)
Supplement: Supplementary file 1 — Supplementary Information [file 41467_2019_14179_MOESM1_ESM.docx]

**Studying biomolecular folding and binding using temperature-jump mass spectrometry**

Adrien Marchand^1^, Martin F. Czar^1^, Elija Eggel^1^, Jérôme Kaeslin^1^, Renato Zenobi^1^

^1^Department of Chemistry and Applied Biosciences, ETH Zurich, CH-8093 Zurich, Switzerland

**List of supplementary figures:**

[Supplementary Figure 1. Picture of the temperature-jump source developed for this work. 2](#_Toc27232537)

[Supplementary Figure 2. Graph of the temperature of the solution in the center of the liquid as a function of the position from the tip, for different flow rates. 3](#_Toc27232538)

[Supplementary Figure 3. Kinetics of formation of the 22CTA G-quadruplex at different temperatures. 4](#_Toc27232539)

[Supplementary Figure 4. Kinetics experiments of 10 µM 22CTA in 100 mM TMAA and 1 mM KCl recorded from a jump from 25 to 75 °C. 5](#_Toc27232540)

[Supplementary Figure 5. Ion mobility spectra of 22CTA without any bound cation showing that the cation binding is intimately linked to the folding of the G-quadruplex and vice versa. 5](#_Toc27232541)

[Supplementary Figure 6. Mass spectra illustrating the absence of degradation due to the heating. 6](#_Toc27232542)

[Supplementary Figure 7. Effect of the flow rate on the detected species. 7](#_Toc27232543)

[Supplementary Figure 8. Thermal denaturation and kinetics of formation of the DK33 duplex at different temperatures. 8](#_Toc27232544)

[Supplementary Figure 9. Thermal denaturation and kinetics of formation of the Carbonic Anhydrase-ligand complexes for two ligands: a) 4-Carboxybenzene sulfonamide and b) Benzene sulfonamide. 9](#_Toc27232545)

[Supplementary Figure 10. Thermal denaturation and kinetics of folding of the Ribonuclease A protein. 10](#_Toc27232546)

[Supplementary Figure 11. Thermal denaturation and kinetics of dissociation of the triple helix formed by a collagen model peptide at different temperatures. 11](#_Toc27232547)

[Supplementary Figure 12. Thermal denaturation experiment of the DNA triplex monitored using circular dichroism. 12](#_Toc27232548)

[Supplementary Figure 13. Kinetics of formation of the DNA duplex and triplex at different temperatures. 13](#_Toc27232549)

[Supplementary Figure 14. Kinetics of formation of the DNA duplex at different temperatures. 14](#_Toc27232550)

[Supplementary Figure 15. Kinetics of formation of the DNA triplex at different temperatures. 15](#_Toc27232551)

[Supplementary Figure 16. Comparison of the enthalpies and entropies of formation of the triplex using CD and MS thermal denaturation experiments and MS kinetics experiments. 17](#_Toc27232552)

[Supplementary Figure 17. Comparison of the enthalpies and entropies of formation of the DK33 duplex using MS thermal denaturation experiments and MS kinetics experiments. 18](#_Toc27232553)

[Supplementary Figure 18. Comparison of the enthalpies and entropies of formation of the 22CTA G-quadruplex using MS thermal denaturation experiments and MS kinetics experiments. 19](#_Toc27232554)

[Supplementary Figure 19. Kinetics of the formation of the DNA duplex at 35 °C jumping from different temperatures. 20](#_Toc27232555)

[Supplementary Figure 20. Effect of the distance between the outlet of the capillary and the inlet of the MS on the kinetics. 21](#_Toc27232556)

[Supplementary Figure 21. Example of ion mobility mass spectrometry 2D map that was used to extract overlapping signals in the *m/z* axis. 22](#_Toc27232557)

[Supplementary Figure 22. Total ion current (TIC) and extracted ion currents for the monomers M_a_ and M_b_, the dimer D and the trimer T. 23](#_Toc27232558)

**List of supplementary tables:**

[Supplementary Table 1. Flow rates, respective times spent in the second block for a 100 µm ID capillary and estimation of the error on the time axis. 2](#_Toc27232570)

[Supplementary Table 2. Rate constants obtained from the fitting of the kinetics recorded at different temperatures for the formation of the DNA duplex and triplex. 16](#_Toc27232571)

[Supplementary Table 3. Comparison of the rate constants from this work with literature values. 16](#_Toc27232572)

**List of supplementary notes:**

[Supplementary Note 1. Commented script used by DynaFit for the fitting. 24](#_Toc27232578)


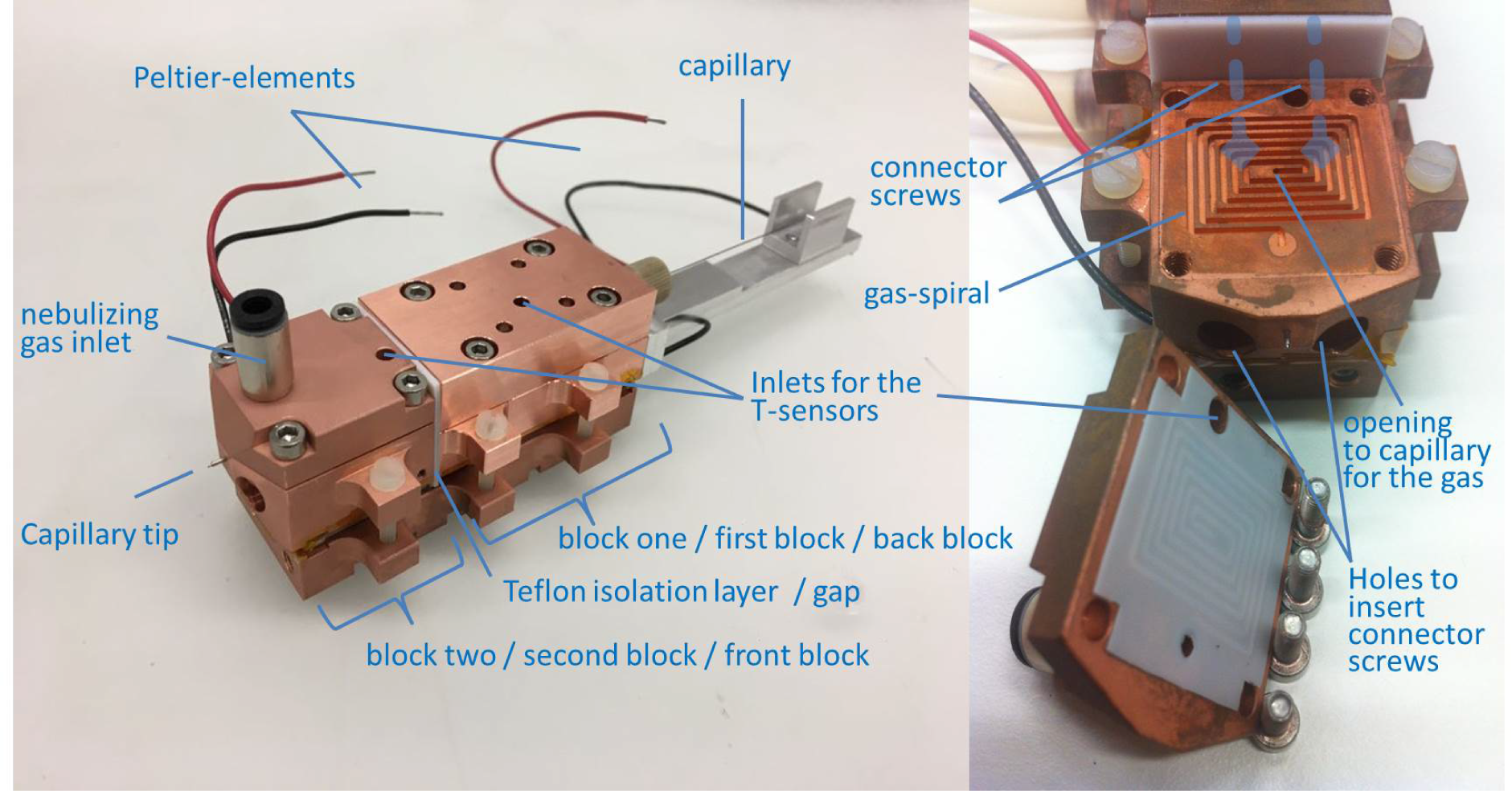


Supplementary Figure 1. Picture of the temperature-jump source developed for this work.

Left, view from outside of the two copper blocks. Right, the front block is opened to show the gas spiral used to pre-heat the coaxial desolvation gas. The main features of the source are annotated.

Supplementary Table 1. Flow rates, respective times spent in the second block for a 100 µm ID capillary and estimation of the error on the time axis.

| **Flow rate (µL/min)** | **Time block2 (s)** | **Time in the 1 mm Teflon gap, *t*_gap_ (s)** | **Remaining distance in block2 for *T* <+1°C of the target value (m)*** | **% of the block used to cool** | **Time used to cool, *t*_cool_ (s)** | **Time error (*t*_gap_ + *t*_cool_) (s)** | **Total % error** |
| --- | --- | --- | --- | --- | --- | --- | --- |
| 0.5 | 32.61 | 0.94 | 0.0341 | 1.6 | 0.51 | 1.45 | 4.5 |
| 0.8 | 20.38 | 0.59 | 0.0340 | 1.8 | 0.36 | 0.95 | 4.7 |
| 1 | 16.30 | 0.47 | 0.0340 | 1.8 | 0.29 | 0.76 | 4.7 |
| 1.5 | 10.87 | 0.31 | 0.0339 | 2.0 | 0.21 | 0.53 | 4.9 |
| 2 | 8.15 | 0.24 | 0.0338 | 2.2 | 0.18 | 0.41 | 5.1 |
| 3 | 5.43 | 0.16 | 0.0338 | 2.4 | 0.13 | 0.29 | 5.3 |
| 4 | 4.08 | 0.12 | 0.0336 | 2.8 | 0.11 | 0.23 | 5.7 |
| 5 | 3.26 | 0.09 | 0.0334 | 3.4 | 0.11 | 0.21 | 6.3 |
| 7.5 | 2.17 | 0.06 | 0.0330 | 4.6 | 0.10 | 0.16 | 7.5 |
| 10 | 1.63 | 0.05 | 0.0326 | 5.8 | 0.09 | 0.14 | 8.7 |
| 15 | 1.09 | 0.03 | 0.0316 | 8.6 | 0.09 | 0.13 | 11.5 |
| 20 | 0.82 | 0.02 | 0.0306 | 11.5 | 0.09 | 0.12 | 14.4 |
| 40 | 0.41 | 0.01 | 0.0270 | 22.0 | 0.09 | 0.10 | 24.9 |
| 60 | 0.27 | 0.01 | 0.0241 | 30.5 | 0.08 | 0.09 | 33.4 |
| 100 | 0.16 | 0.005 | 0.0191 | 44.9 | 0.07 | 0.08 | 47.7 |

*Obtained from Comsol Multiphysics simulations


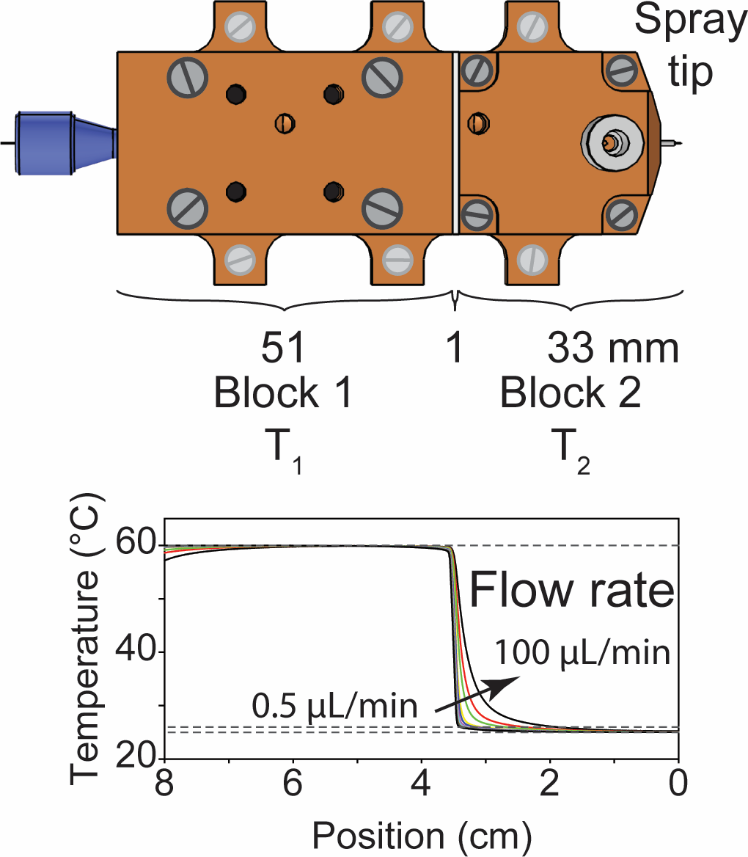


Supplementary Figure 2. Graph of the temperature of the solution in the center of the liquid as a function of the position from the tip, for different flow rates.

A scheme of the source is aligned with the graph. The distance needed to cool the solution increases with the flow rate (See Supplementary Table1 for the values).


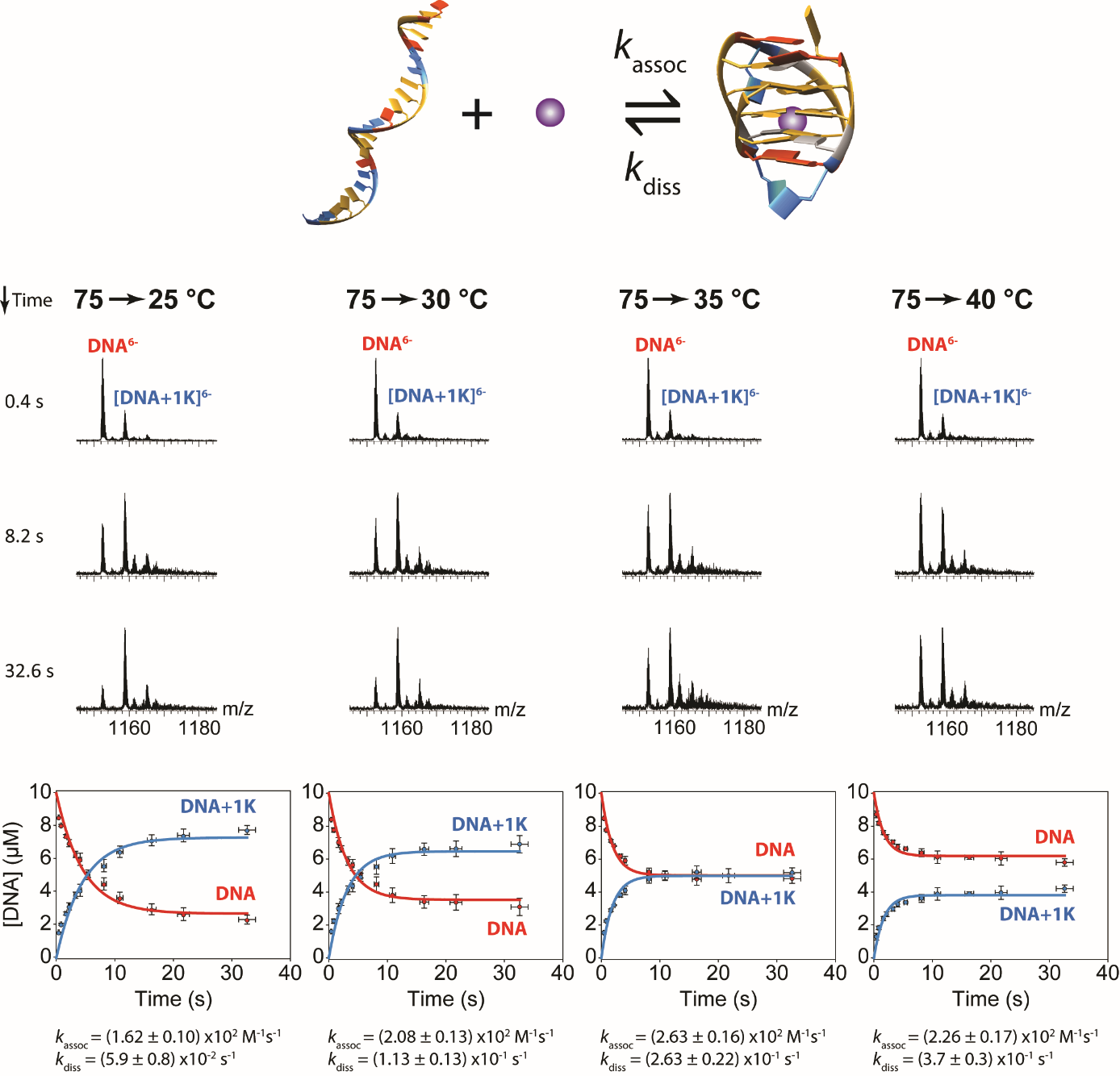


Supplementary Figure 3. Kinetics of formation of the 22CTA G-quadruplex at different temperatures.

Conditions: 10 µM 22CTA in 100 mM TMAA and 1 mM KCl. Top, scheme of the chemical reaction monitored. Middle, representative mass spectra of the kinetics. Bottom, quantification and fitting.

Source data are provided as a Source Data file.


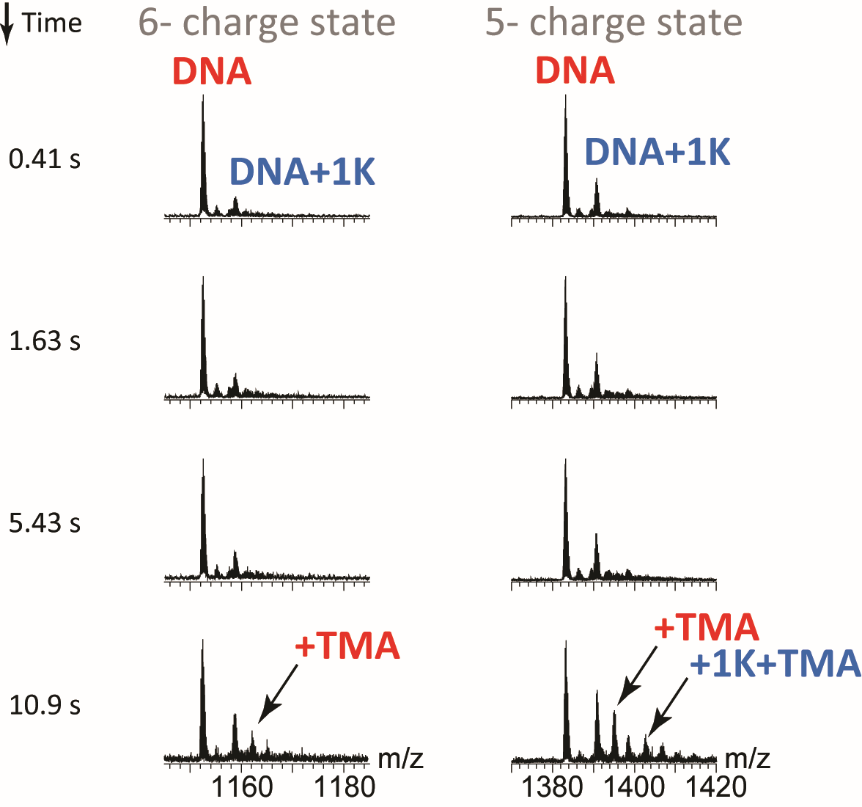


Supplementary Figure 4. Kinetics experiments of 10 µM 22CTA in 100 mM TMAA and 1 mM KCl recorded from a jump from 25 to 75 °C.

Zooms on the 6 and 5- charge states. The distribution of bound cations does not change with the flow rates, indicating that the equilibrium is reached in less than 0.16 s. The peak corresponding to the DNA strand bound to one K^+^ is typical of a nonspecific adduct and was considered as such for the quantification. At low flow rates, two additional adducts corresponding to a TMA adduct on each species, DNA and [DNA+1K], appeared at low flow rates.


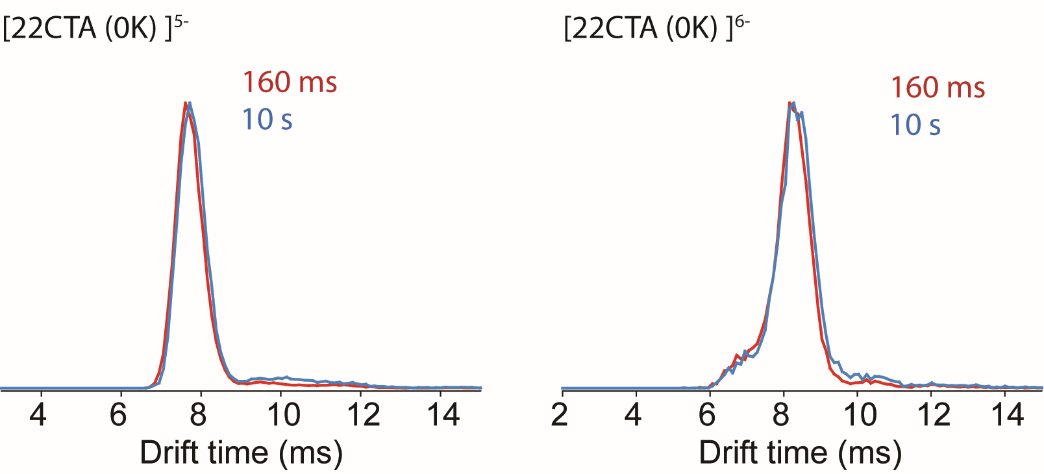


Supplementary Figure 5. Ion mobility spectra of 22CTA without any bound cation showing that the cation binding is intimately linked to the folding of the G-quadruplex and vice versa.

Conditions: 10 µM 22CTA in 100 mM TMAA and 1 mM KCl. After a temperature jump from 25 to 75 °C.

The mobility of the 0-K^+^ species is the same after 160 ms or 10 s at 75 °C. This is in agreement with the fact that, for G-quadruplexes, cation binding is intimately linked to folding and vice versa. This is expected because the CD data (based on the stacking of the bases and therefore the “folding”) and the MS data (based on the number of bound K+) always give similar thermal denaturation temperatures.


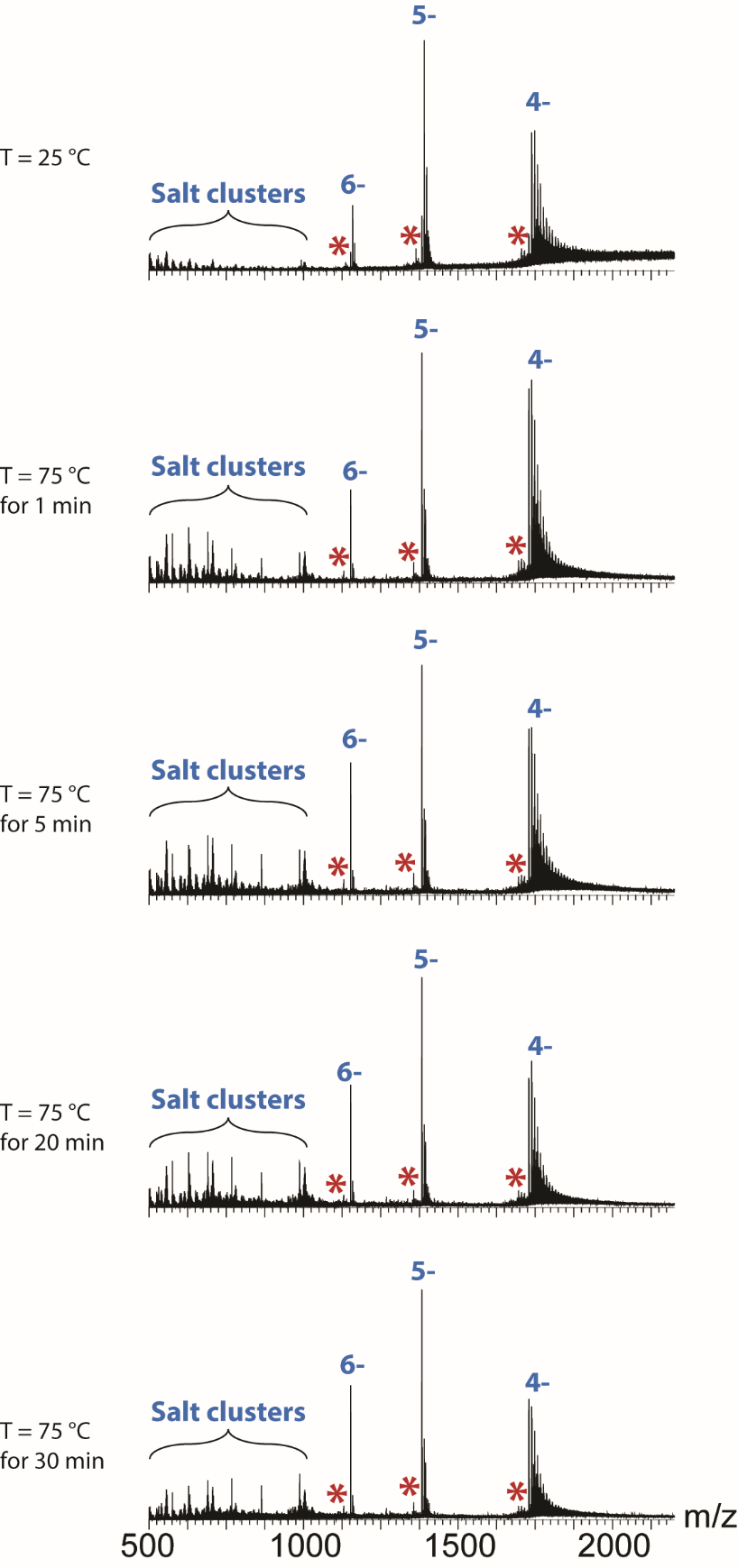


Supplementary Figure 6. Mass spectra illustrating the absence of degradation due to the heating.

Conditions: 10 µM 22CTA in 100 mM TMAA and 1 mM KCl.

The Supplementary Figurehows that even after heating the solution for up to 30 min, the whole mass spectra remain very similar. We note that a peak that could correspond to degradation was observed and correspond to a base loss (annotated with a red star). This degradation is however not due to the heating because it is also present at 25 °C in the sample that was not heated (top). At low mass, salt clusters are observed as well as peaks of low intensity corresponding to higher charge states of the DNA. No trace of degradation was observed.


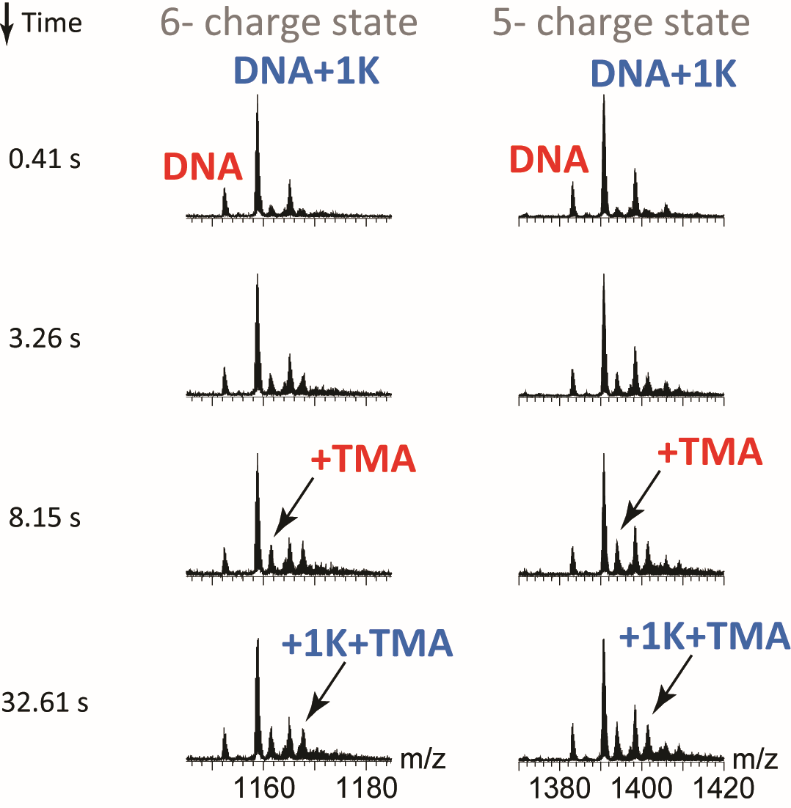


Supplementary Figure 7. Effect of the flow rate on the detected species.

Conditions: 10 µM 22CTA in 100 mM TMAA and 1 mM KCl at 25 °C. Zooms on the 6 and 5- charge states. The distribution of bound cations does not change with the flow rates. Two additional adducts corresponding to a TMA adduct on each species, DNA and [DNA+1K], appeared at low flow rates.


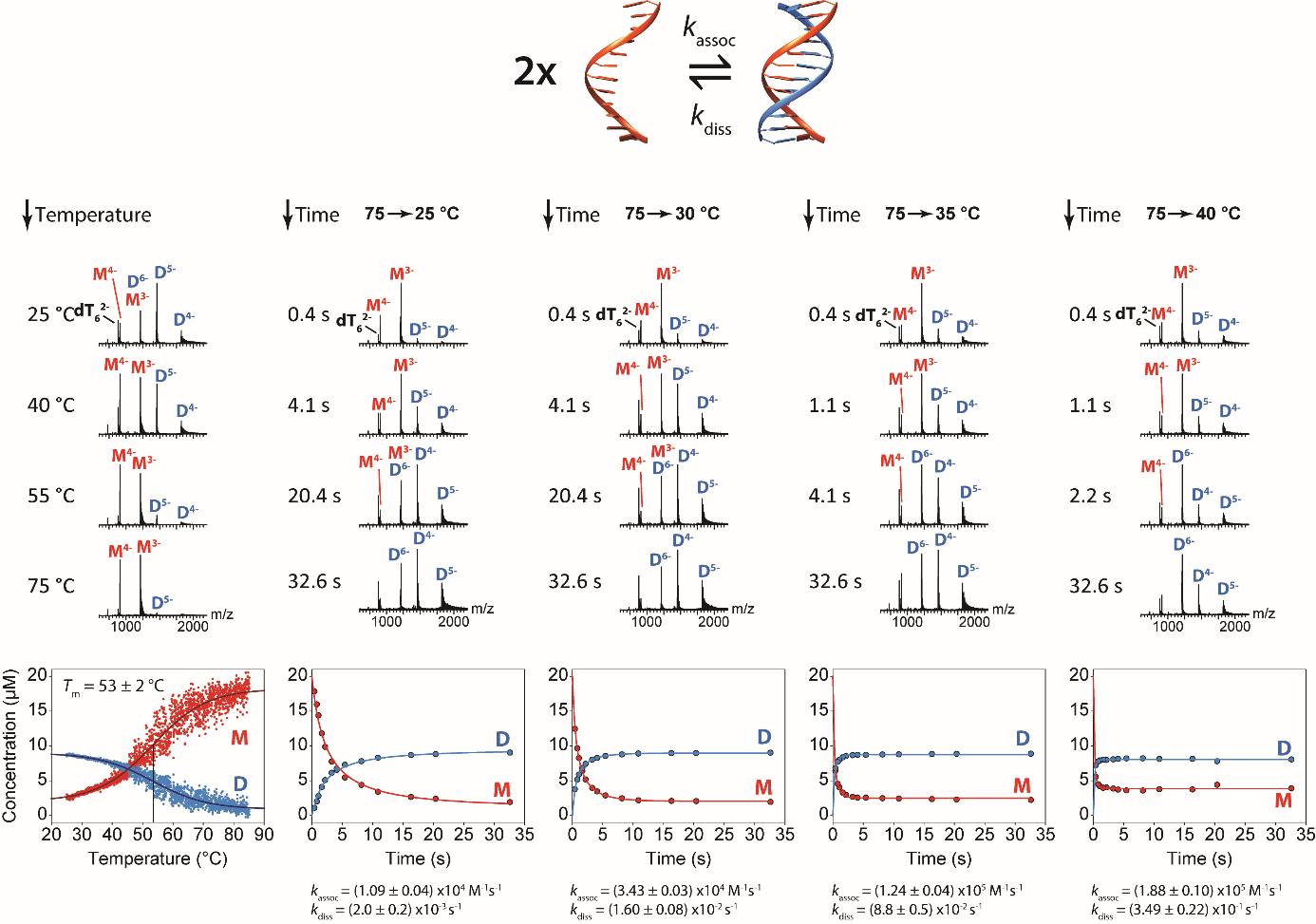


Supplementary Figure 8. Thermal denaturation and kinetics of formation of the DK33 duplex at different temperatures.

Conditions: 20 µM DK33 in 100 mM TMAA. Top, scheme of the chemical reaction monitored. Middle, representative mass spectra of the thermal denaturation and kinetics. Bottom, quantification and fitting.

The errors on the rate constants are the standard deviations from the fitting.


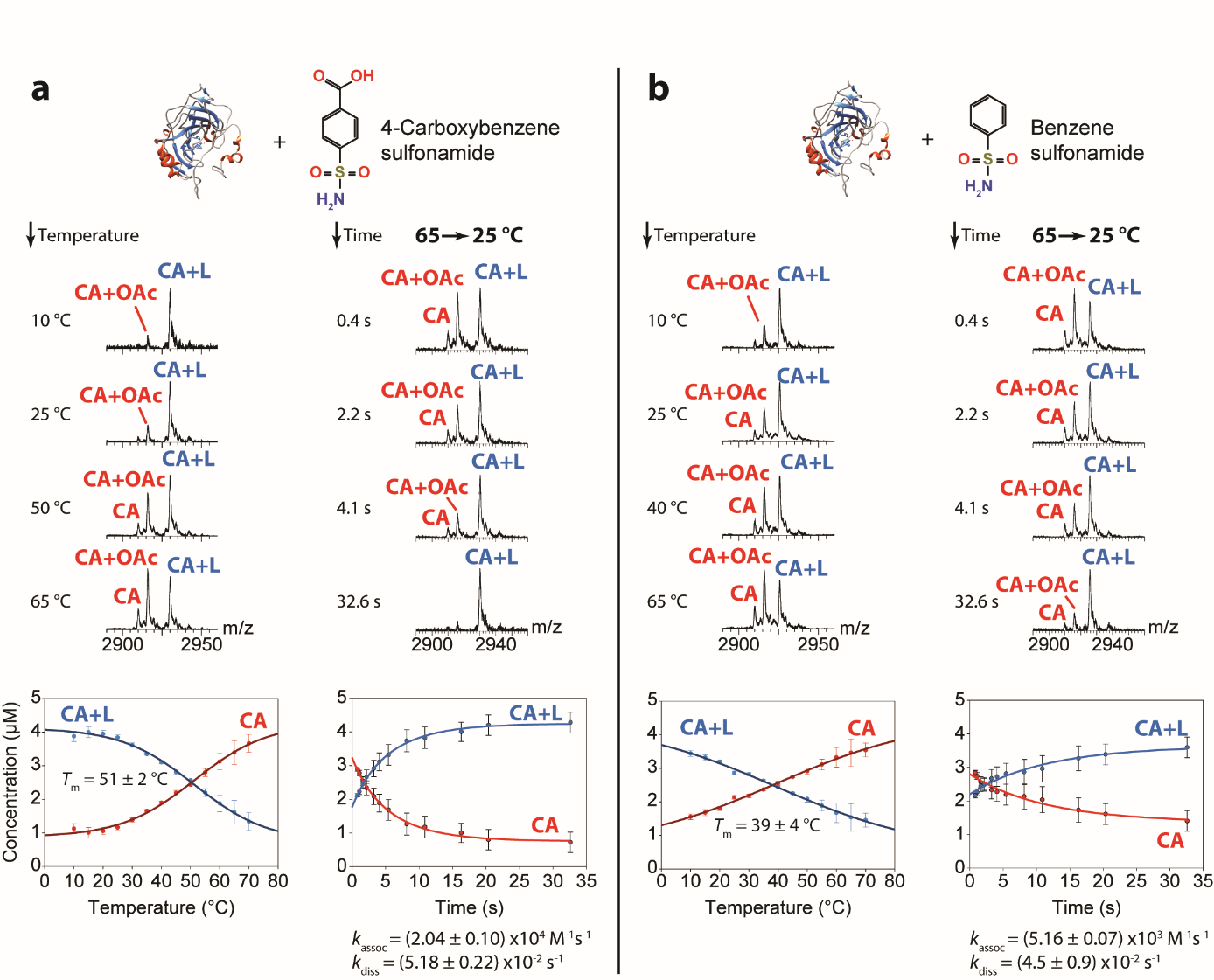


Supplementary Figure 9. Thermal denaturation and kinetics of formation of the Carbonic Anhydrase-ligand complexes for two ligands: a) 4-Carboxybenzene sulfonamide and b) Benzene sulfonamide.

Conditions: 5 µM Carbonic Anhydrase with 5 µM ligand in 10 mM NH_4_OAc. Zooms on the 10+ charge state. The error bars represent the standard deviation between the charge states used for quantification (9, 10, 11 and 12+ charge states). Top, scheme of interacting partners. Middle, representative mass spectra of the thermal denaturation and kinetics. Bottom, quantification and fitting.

The errors on the rate constants are the standard deviations from the fitting. Source data are provided as a Source Data file.


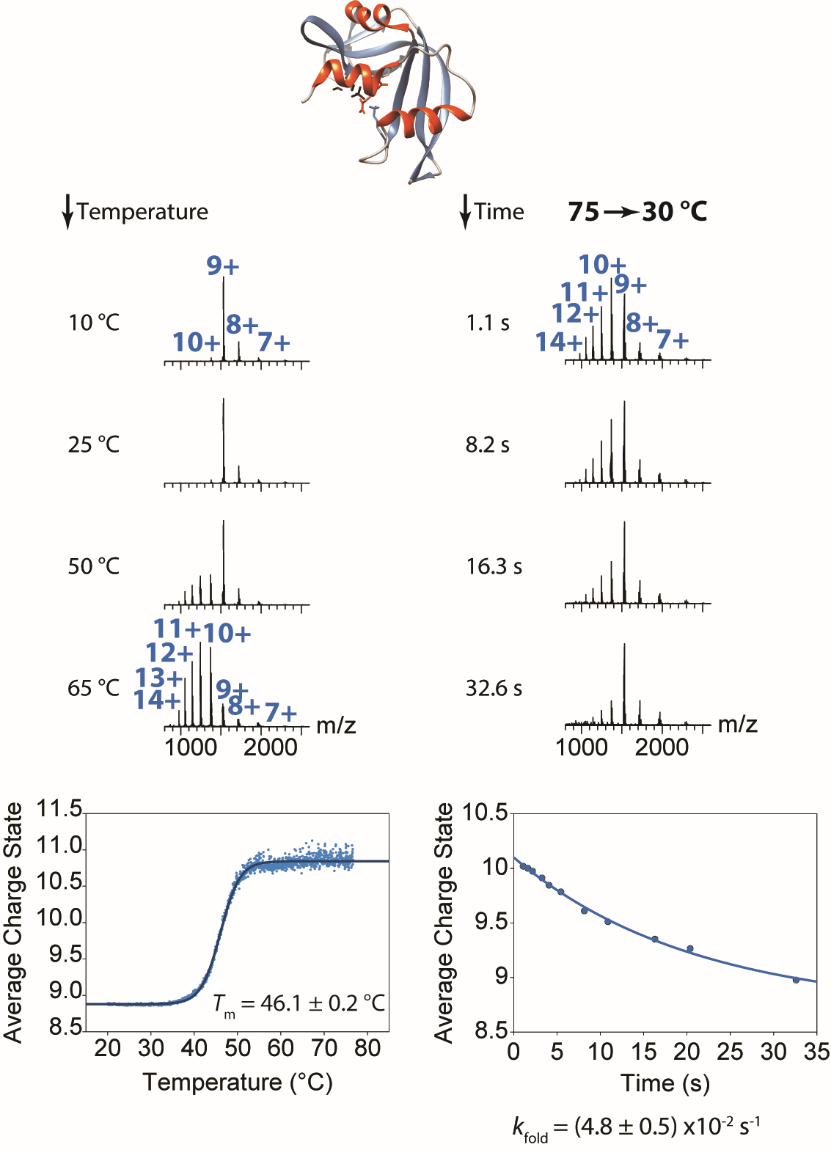


Supplementary Figure 10. Thermal denaturation and kinetics of folding of the Ribonuclease A protein.

Conditions: 10 µM protein in 100 mM NH_4_OAc at pH 2.75. The average charge state was calculated as a function of temperature or time. Top, scheme of Ribonuclease A. Middle, representative mass spectra of the thermal denaturation and kinetics. Bottom, quantification and fitting.

The errors on the rate constants are the standard deviations from the fitting.


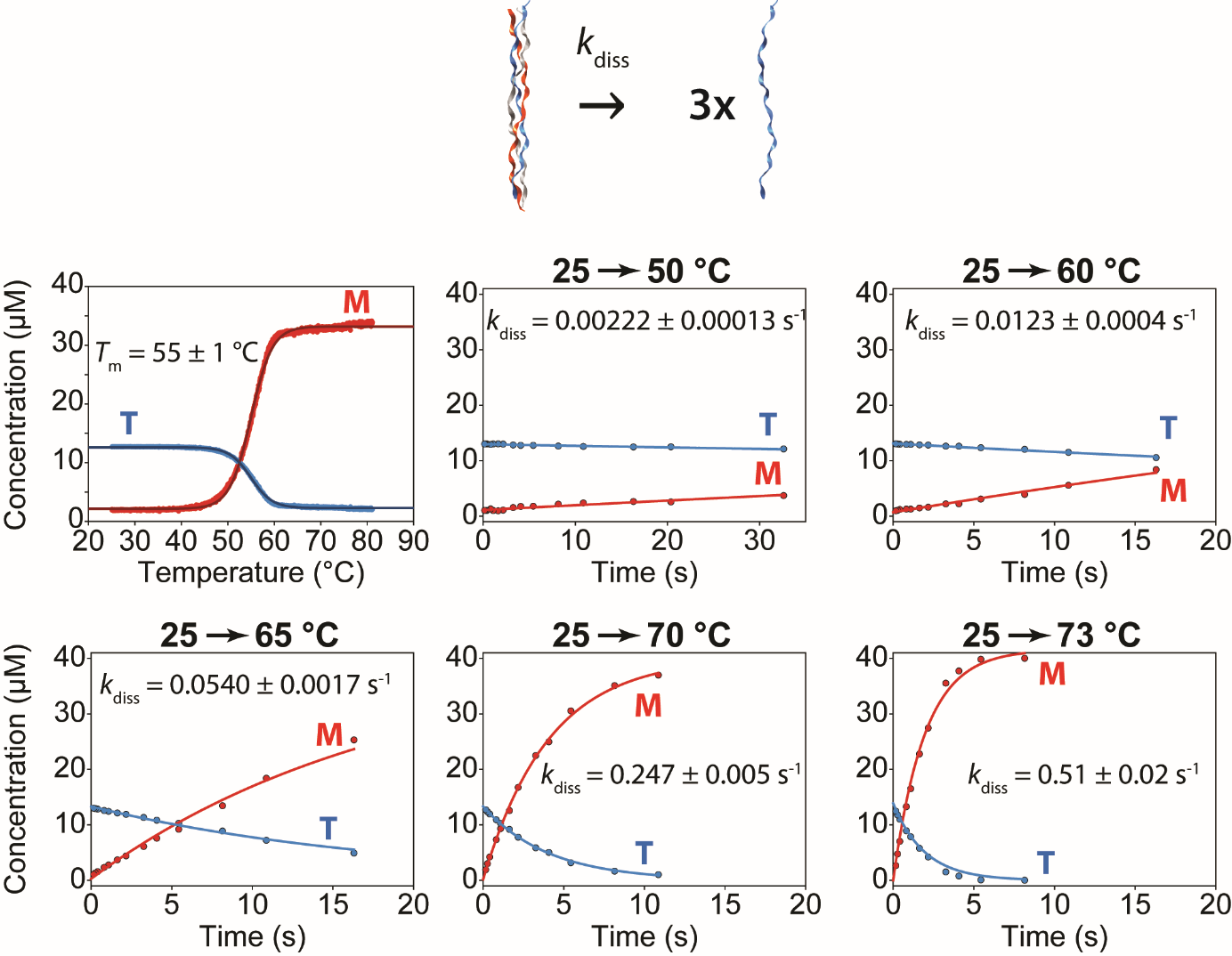


Supplementary Figure 11. Thermal denaturation and kinetics of dissociation of the triple helix formed by a collagen model peptide at different temperatures.

Peptide: [Proline-Hydroxyproline-Glycine]_8_, [POG]_8_; PDB ID: 1CGD; Conditions: 40 µM [POG]_8_ in 10 mM NH_4_OAc. Top, scheme of the monitored reaction. Bottom, quantification and fitting.

The errors on the rate constants are the standard deviations from the fitting.


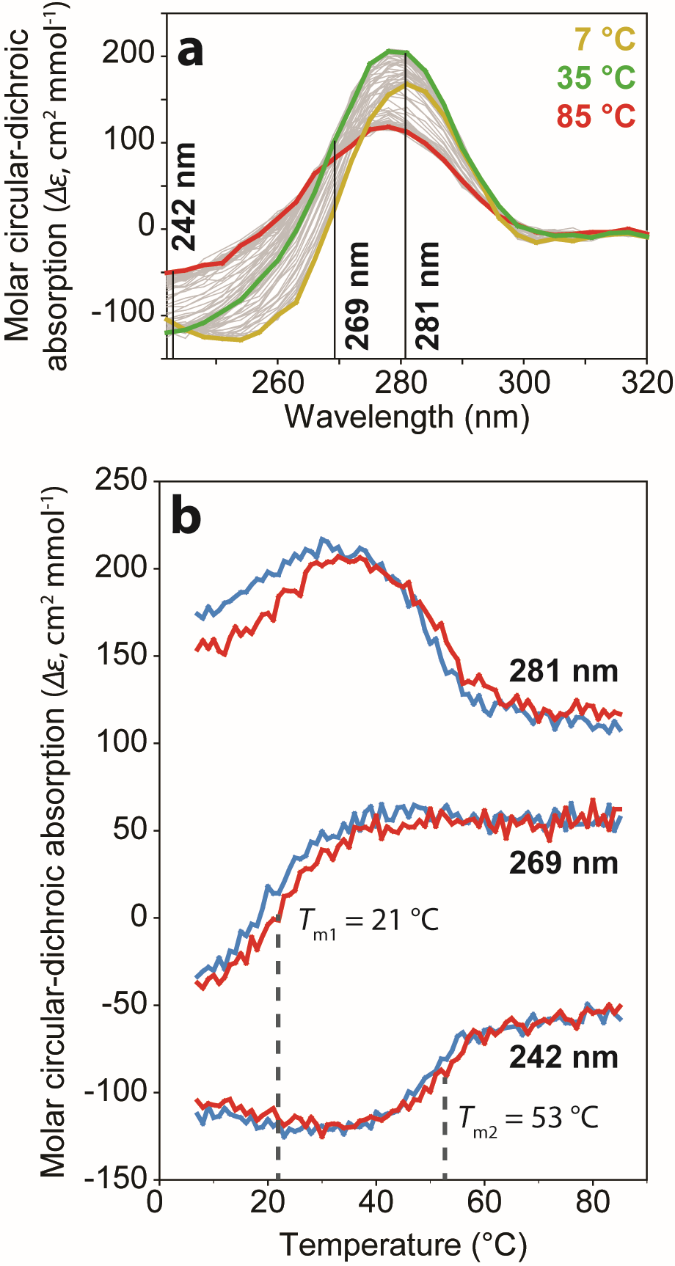


Supplementary Figure 12. Thermal denaturation experiment of the DNA triplex monitored using circular dichroism.

Conditions: 10 µM M_a_, 10 µM M_b_ and 30 µM M_c_ in 100 mM TMAA at pH 5.5. a) CD spectra recorded during the thermal denaturation experiment. b) Molar circular-dichroic absorption at different wavelengths as a function of temperature, heating from 7 to 85 °C in red and cooling from 85 to 7 °C in blue.


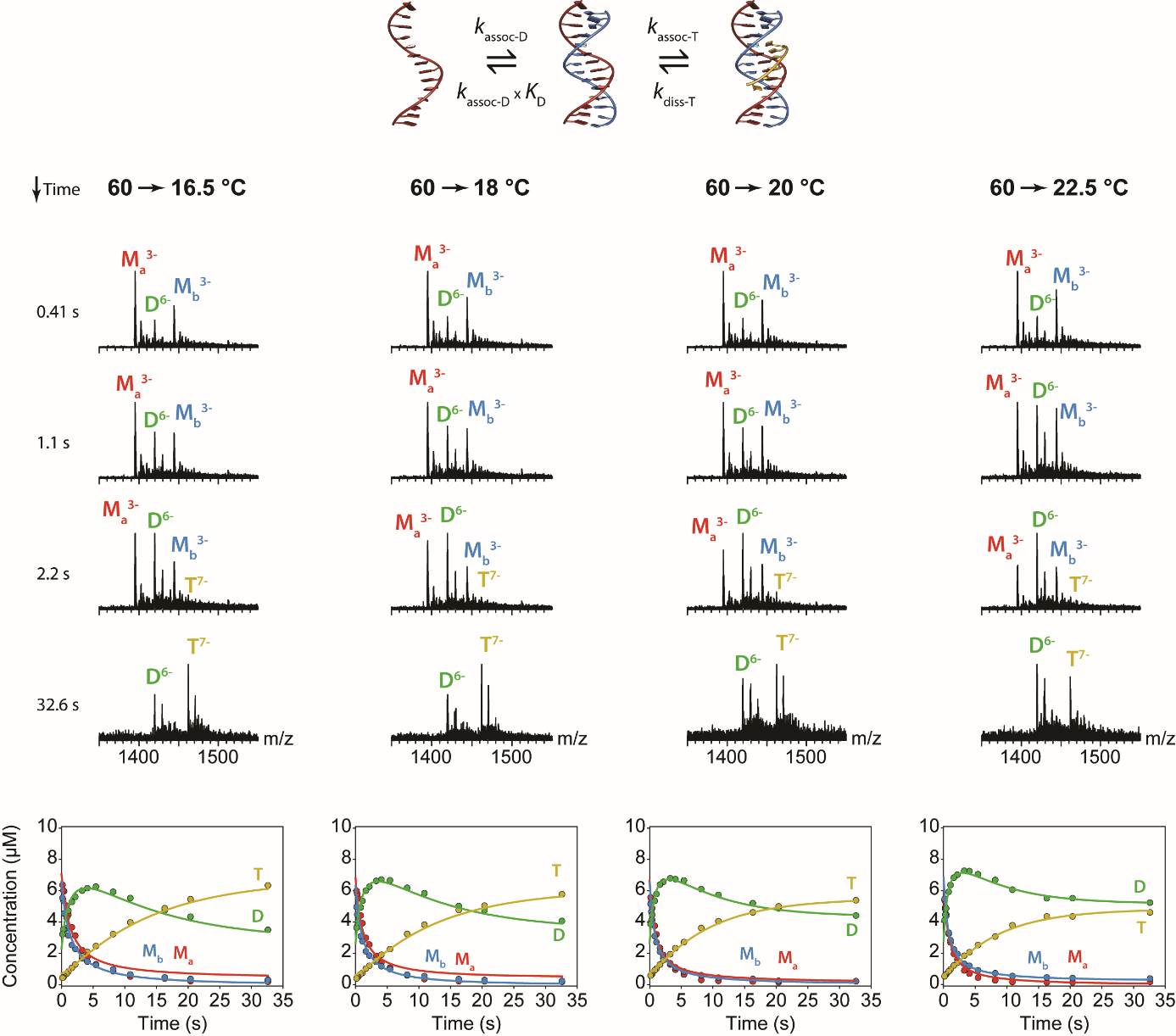


Supplementary Figure 13. Kinetics of formation of the DNA duplex and triplex at different temperatures.

Conditions: 10 µM M_a_, 10 µM M_b_ and 30 µM M_c_ in 100 mM TMAA at pH 5.5. Top, scheme of the monitored reactions. Middle, representative mass spectra of the kinetics. Bottom, quantification and fitting.


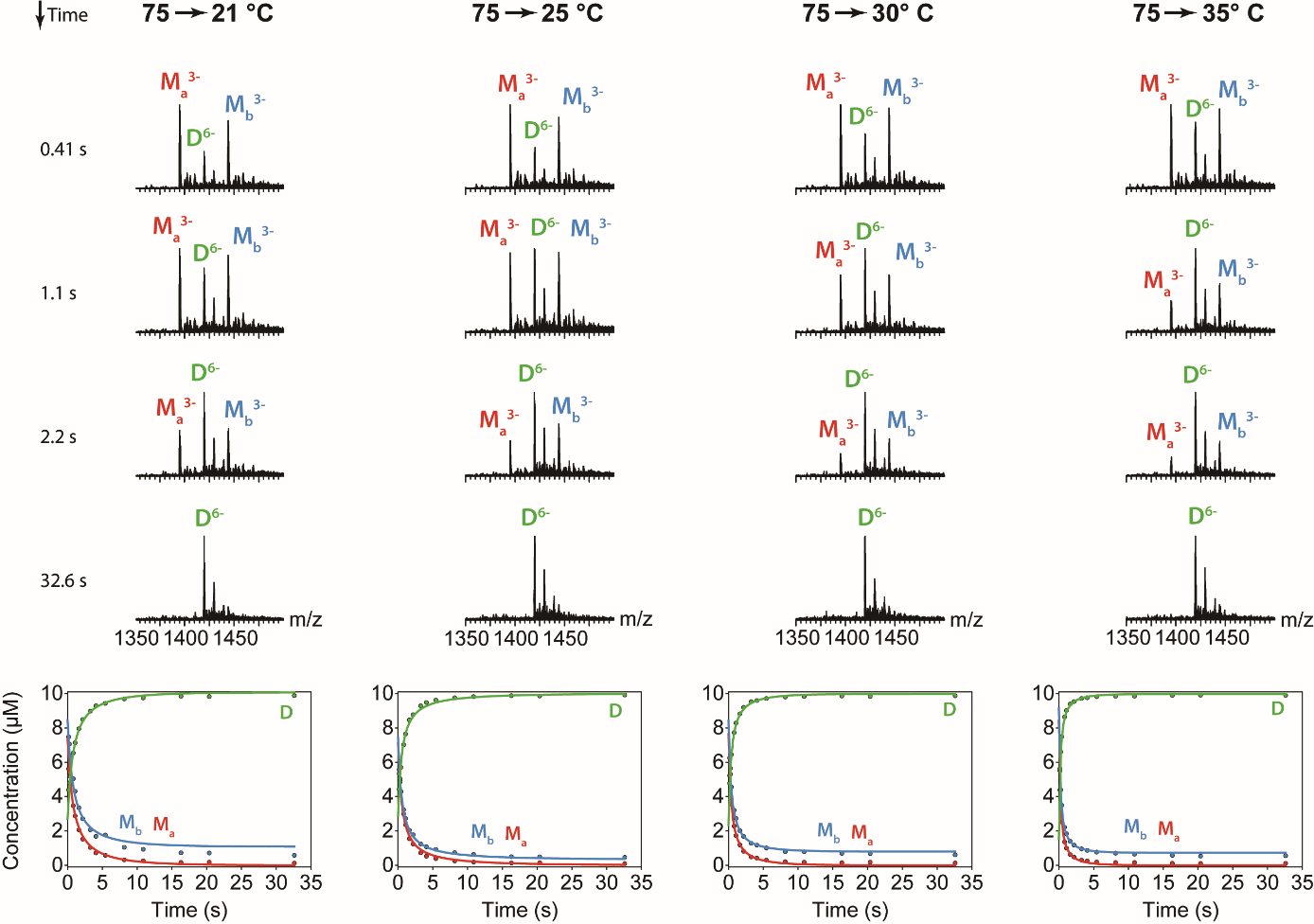


Supplementary Figure 14. Kinetics of formation of the DNA duplex at different temperatures.

Conditions: 10 µM M_a_, 10 µM M_b_ in 100 mM TMAA at pH 5.5. Top, representative mass spectra of the kinetics. Bottom, quantification and fitting.


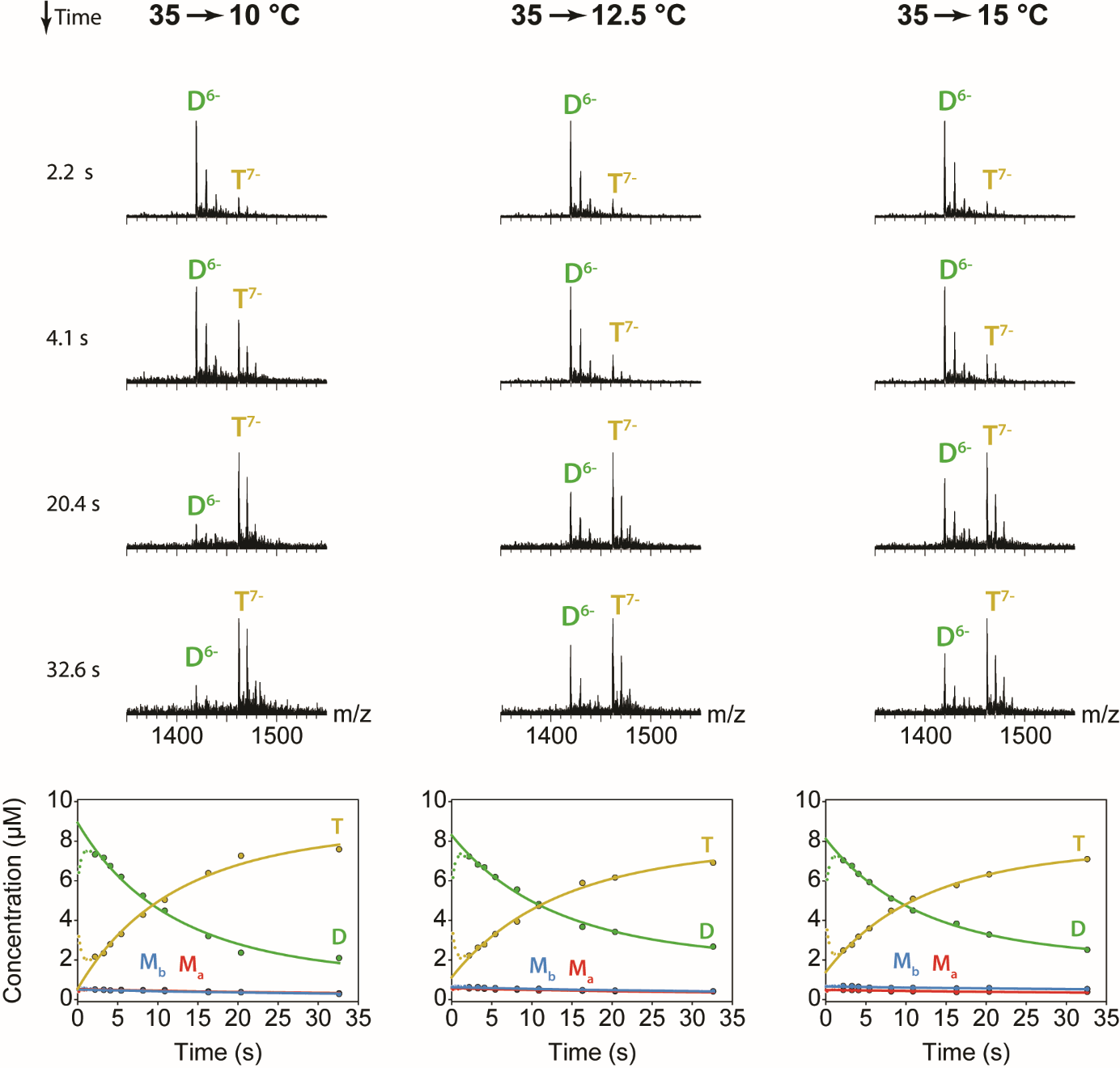


Supplementary Figure 15. Kinetics of formation of the DNA triplex at different temperatures.

Conditions: 10 µM M_a_, 10 µM M_b_ and 30 µM M_c_ in 100 mM TMAA at pH 5.5. The points below 2 s reaction time (represented as smaller dots) were not included for the fitting because the equilibrium in the first block was not yet reached. Top, representative mass spectra of the kinetics. Bottom, quantification and fitting.

Supplementary Table 2. Rate constants obtained from the fitting of the kinetics recorded at different temperatures for the formation of the DNA duplex and triplex.

The errors on the rate constants are the standard deviations from the fitting.

| *T* (°C) | *k*_assoc-D_  (x10^5^ M^-1^s^-1^) | *k*_assoc-T_  (x10^3^ M^-1^s^-1^) | *k*_diss-T_  (x10^-2^ s^-1^) |
| --- | --- | --- | --- |
| 10 | N.D. | 2.7 ± 0.2 | 0.73 ± 0.22 |
| 12.5 | N.D. | 2.18 ± 0.09 | 1.22 ± 0.16 |
| 15 | N.D. | 2.33 ± 0.06 | 1.41 ± 0.10 |
| 16.5 | 1.07 ± 0.06 | 2.06 ± 0.18 | 2.1 ± 0.6 |
| 18 | 1.41 ± 0.08 | 1.94 ± 0.17 | 2.7 ± 0.6 |
| 20 | 1.80 ± 0.07 | 2.36 ± 0.15 | 4.7 ± 0.5 |
| 21 | 1.51 ± 0.07 | N.D. | N.D. |
| 22.5 | 2.01 ± 0.06 | 2.22 ± 0.10 | 6.1 ± 0.5 |
| 25 | 2.44 ± 0.08 | N.D. | N.D. |
| 30 | 3.13 ± 0.07 | N.D. | N.D. |
| 35 | 4.5 ± 0.5 | 6.4 ± 0.5 | 133 ± 8 |

Supplementary Table 3. Comparison of the rate constants from this work with literature values.

|  |  | Value  (this work) | Literature value | Reference | Conditions (this work) | Conditions (literature) |
| --- | --- | --- | --- | --- | --- | --- |
| CA + 4-Cbxbz | ***k*_assoc_** | 2.04 x 10^4^ M^-1^ s^-1^ | 2.58 x 10^4^ M^-1^ s^-1^ | ^[1]^ | 10 mM | 50 mM |
| sulfonamide | ***k*_diss_** | 5.18 x 10^-2^ s^-1^ | 7.5 x 10^-2^ s^-1^ |  | NH_4_OAc | NH_4_OAc |
| CA + | ***k*_assoc_** | 5.16 x 10^3^ M^-1^ s^-1^ | 9.18 x 10^3^ M^-1^ s^-1^ | ^[1]^ | 10 mM | 50 mM |
| Bzsulfonamide | ***k*_diss_** | 4.5 x 10^-2^ s^-1^ | 1.025 x 10^-2^ s^-1^ |  | NH_4_OAc | NH_4_OAc |
| RNase A | ***k*_fold_** | 4.8 x 10^-2^ s^-1^ | 2.8 x 10^-2^ s^-1^ | ^[2]^ | 30 °C | 29 °C |

The table highlights the differences in the conditions in which the values where measured.


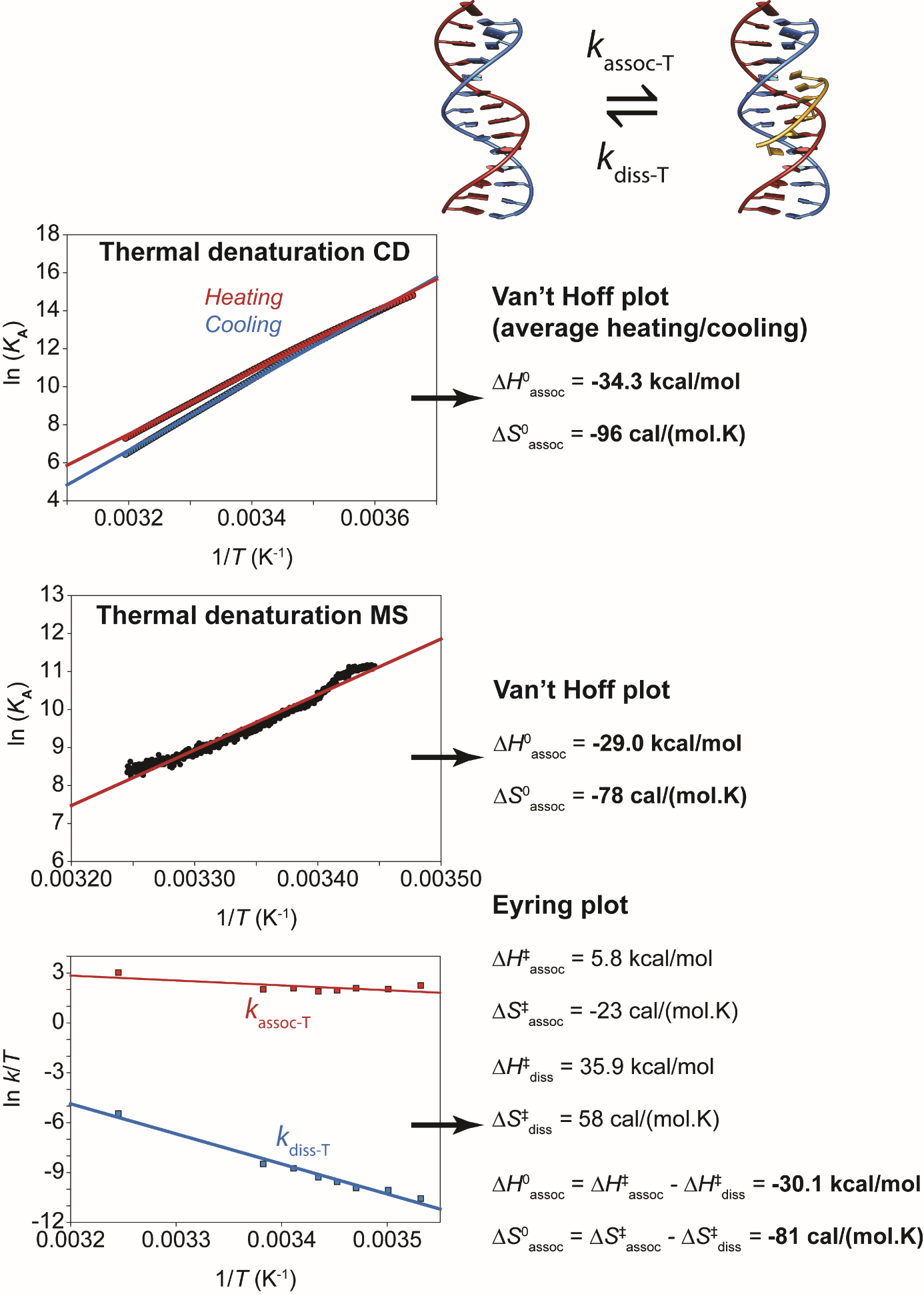


Supplementary Figure 16. Comparison of the enthalpies and entropies of formation of the triplex using CD and MS thermal denaturation experiments and MS kinetics experiments.


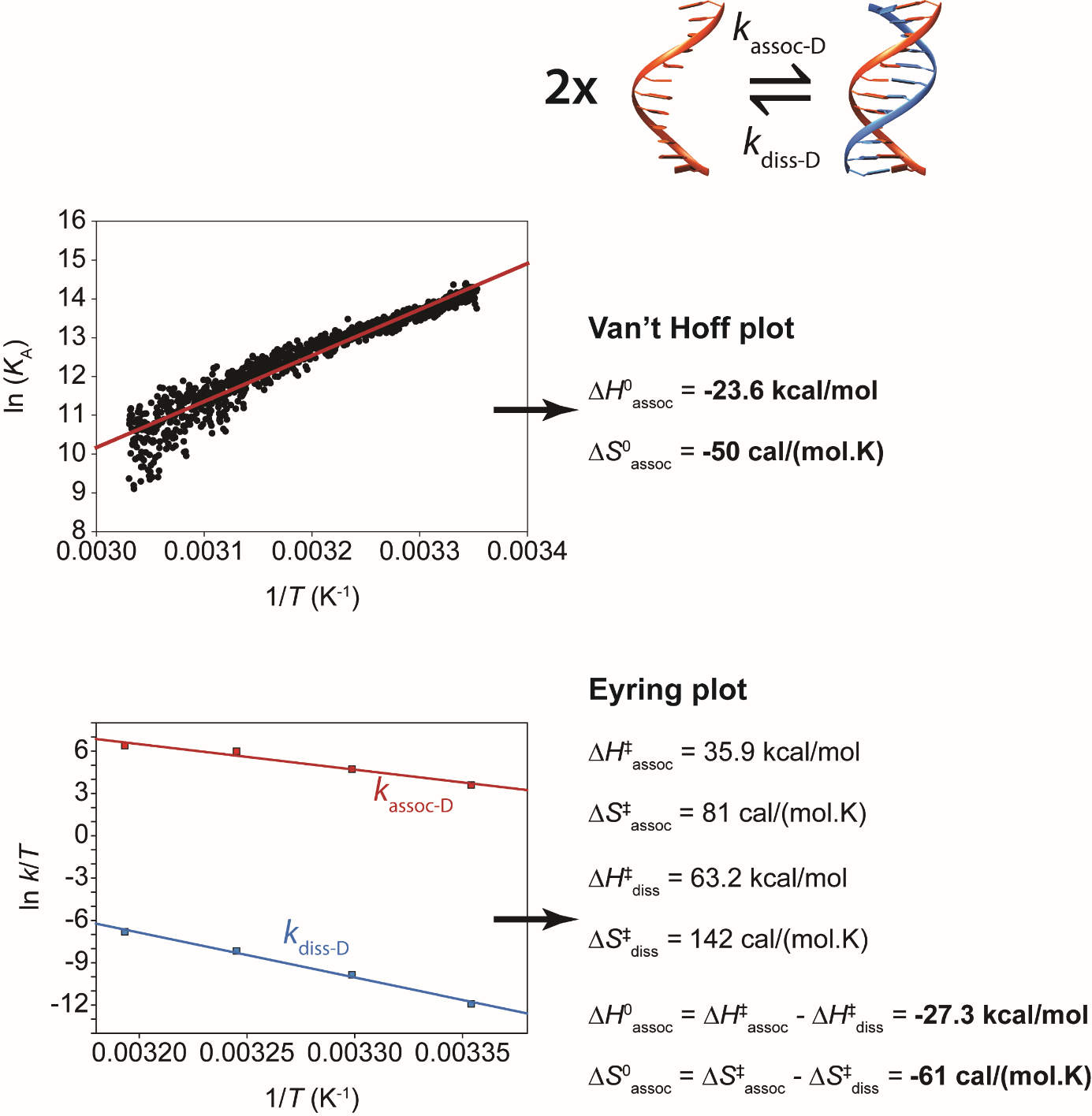


Supplementary Figure 17. Comparison of the enthalpies and entropies of formation of the DK33 duplex using MS thermal denaturation experiments and MS kinetics experiments.


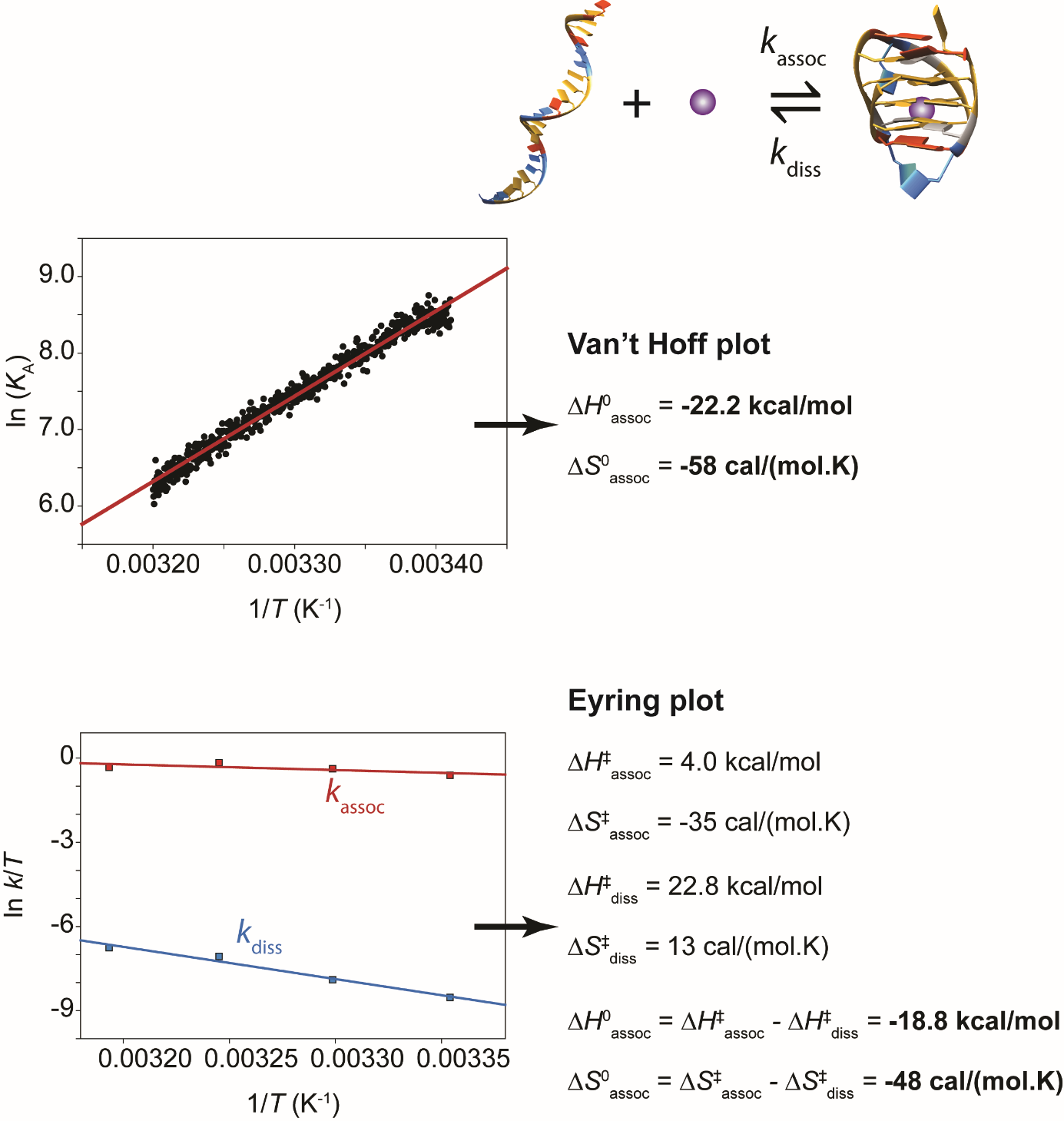


Supplementary Figure 18. Comparison of the enthalpies and entropies of formation of the 22CTA G-quadruplex using MS thermal denaturation experiments and MS kinetics experiments.


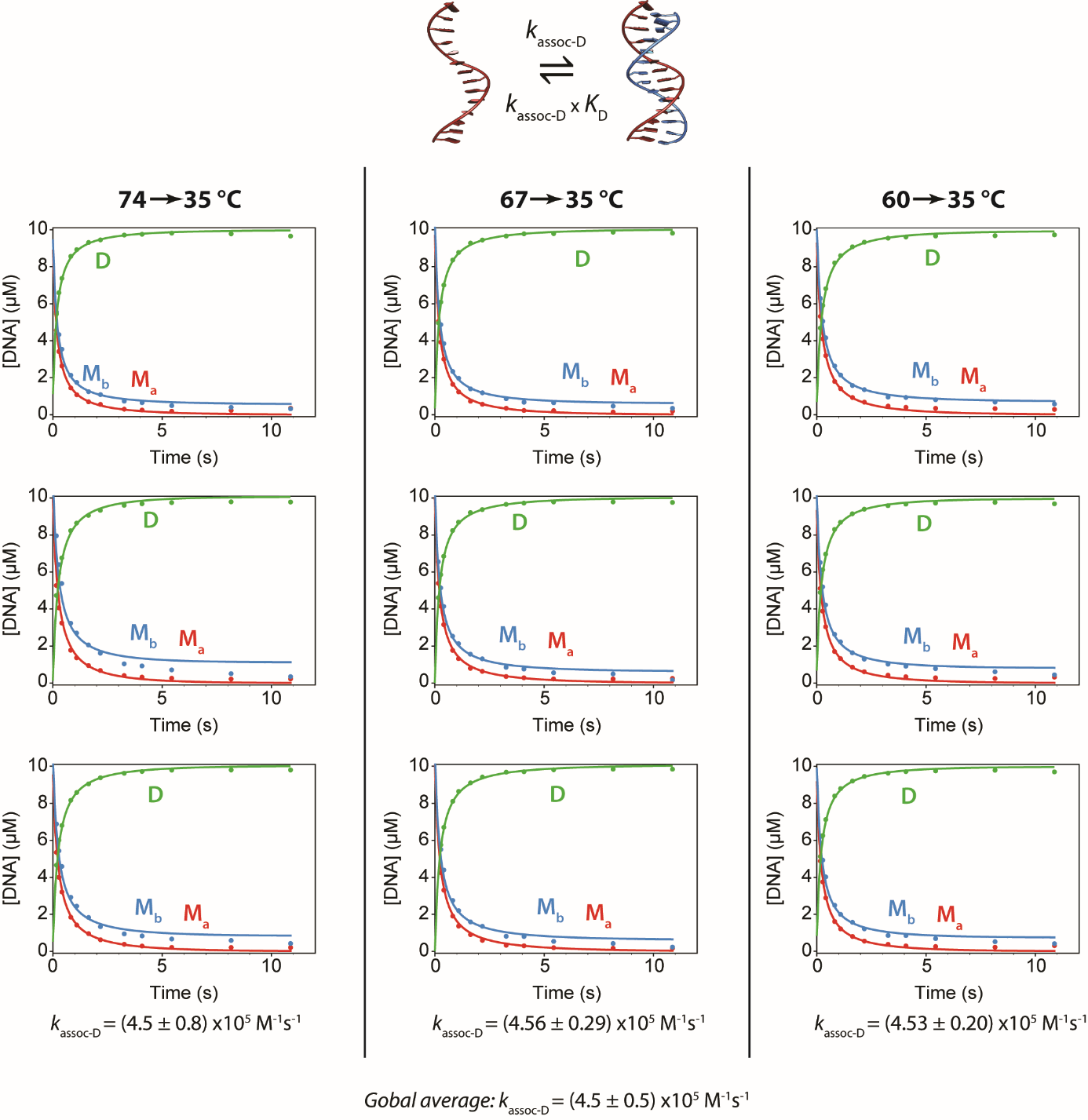


Supplementary Figure 19. Kinetics of the formation of the DNA duplex at 35 °C jumping from different temperatures.

Conditions: 10 µM M_a_, 10 µM M_b_ in 100 mM TMAA at pH 5.5. Top, scheme of the monitored reaction. Bottom, quantification and fitting of three different experiments, each performed from three different starting temperature.

The errors on the rate constants are the standard deviations from the fitting.


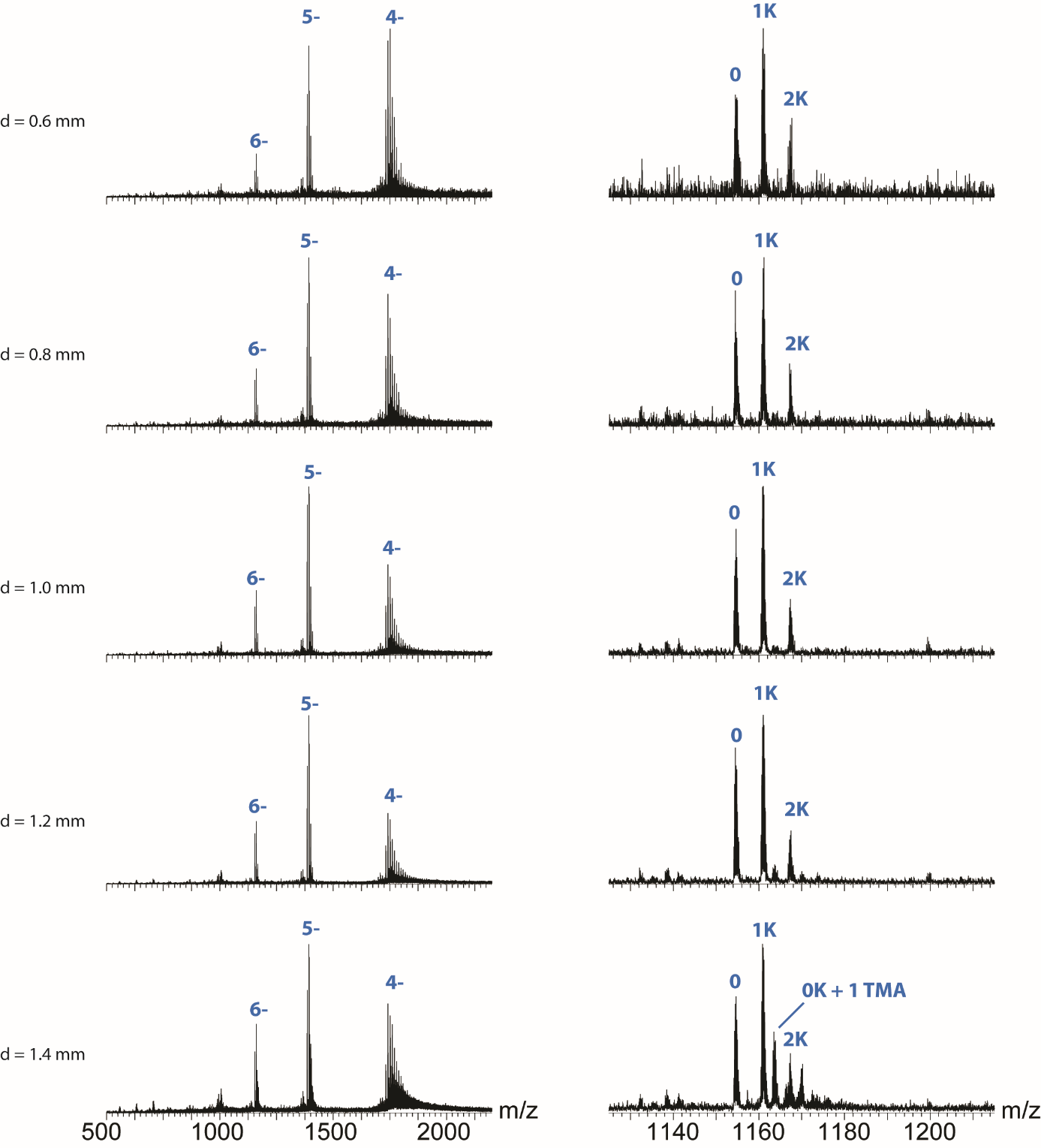


Supplementary Figure 20. Effect of the distance between the outlet of the capillary and the inlet of the MS on the kinetics.

Conditions: 10 µM 22CTA in 100 mM TMAA and 1 mM KCl. Recorded after a temperature-jump from 75 to 25 °C at different distances. The flow rate was fixed to 3 µL/min.

The Supplementary Figurehows how the recorded mass spectra change with the position of the capillary in from of the MS. At short distances, the signal is weaker. At long distances, more TMA adducts are observed. Overall, the proportions of the 0, 1 and 2-K^+^ stoichiometries appear constant throughout all the tested ranges.


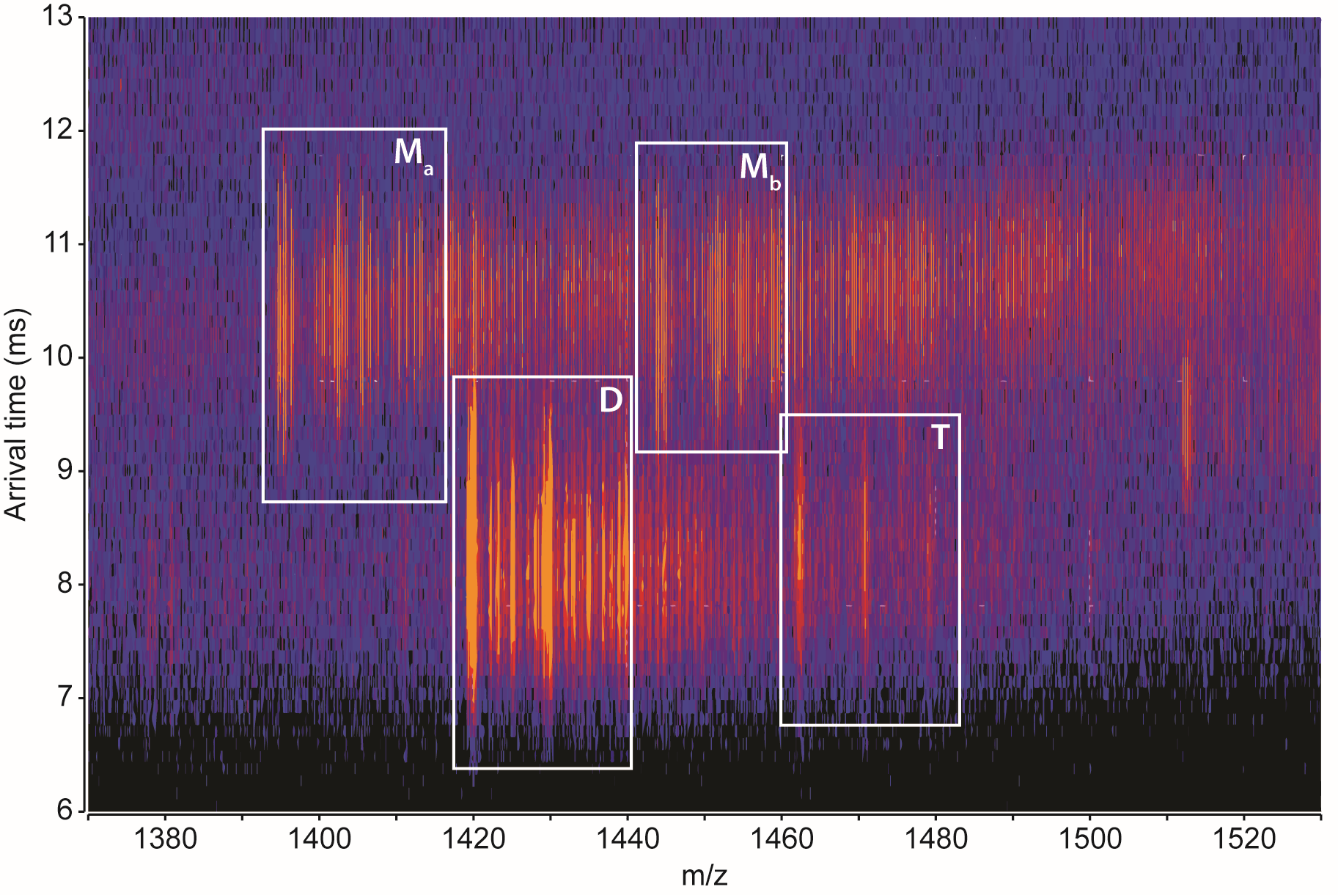


Supplementary Figure 21. Example of ion mobility mass spectrometry 2D map that was used to extract overlapping signals in the *m/z* axis.

In this case, a zoom on the monomers 3-, dimer 6- and trimer 7- is displayed for a solution containing 10 µM M_a_ and M_b_, 30 µM M_c_ in 100 mM TMAA at pH 5.5. The white squares correspond to the areas that were typically integrated. The other peaks are completely separated on the *m/z* axis and are not displayed on this graph, but were used for the quantification.


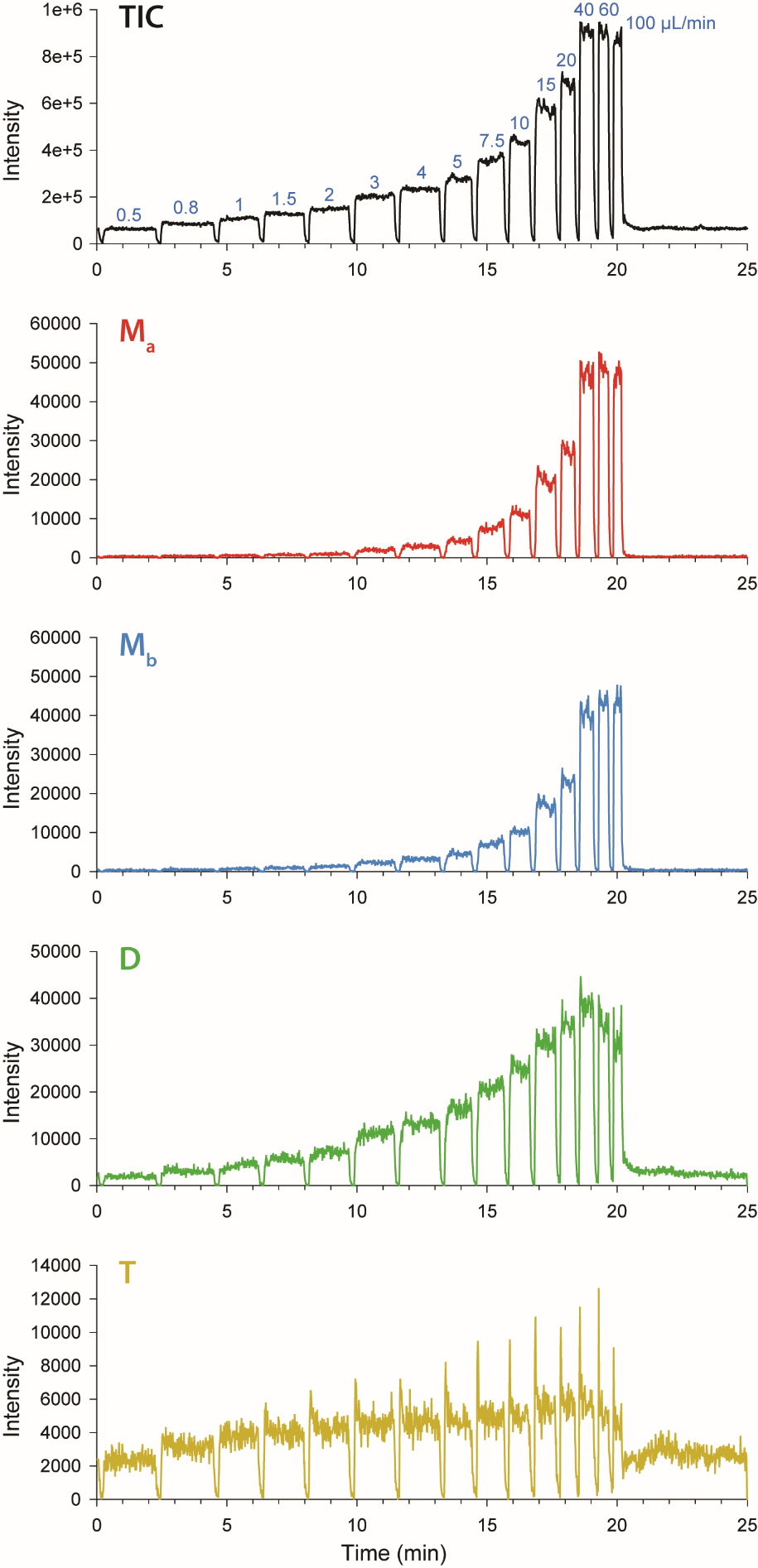


Supplementary Figure 22. Total ion current (TIC) and extracted ion currents for the monomers M_a_ and M_b_, the dimer D and the trimer T.

Experiment corresponding to a temperature jump from 60 to 25 °C for a solution containing 10 µM M_a_ and M_b_, 30 µM M_c_ in 100 mM TMAA at pH 5.5. The flow rates are indicated in blue on the top panel. More intense signals are obtained when higher flow rates are used.

Supplementary Note 1. Commented script used by DynaFit for the fitting.

In black, the used script and in blue, the comments. Script used for the jump from 60 to 20 °C for the formation of the triplex.

[task]

data = progress

task = fit

[mechanism]

M_a_ + M_b_ <==> D : k_assoc-D_ k_diss-D_

D + M_c_ <==> T : k_assoc-T_ k_diss-T_

[constants]

k_assoc-D_ = 0.1 ?, k_diss-D_ = 5.60494E-10 * k_assoc-D_

k_assoc-T_ = 0.1 ?, k_diss-T_ = 0.1 ?

Definition of the chemical mechanism and of the rate constants. The question mark after a value indicates that the rate will be adjusted. In this example, the chemical mechanism and the kinetics differential equations are the following:

$$M_{A}+M_{B}+M_{C} \begin{matrix} k_{assoc-D} \\ \rightleftharpoons\\ k_{diss-D} \end{matrix} D+M_{C} \begin{matrix} k_{assoc-T} \\ \rightleftharpoons\\ k_{diss-T} \end{matrix} T$$

$$\frac{d{[M}_{A}]}{\mathrm{dt}}=-k_{assoc-D} {[M}_{A}] {[M}_{B}]+k_{diss-D} [D]$$

$$\frac{d{[M}_{B}]}{\mathrm{dt}}=-k_{assoc-D} {[M}_{A}] {[M}_{B}]+k_{diss-D} [D]$$

$$\frac{d{[M}_{C}]}{\mathrm{dt}}=-k_{assoc-T} [D] {[M}_{C}]+k_{diss-T} [T]$$

$$\frac{d[D]}{\mathrm{dt}}=k_{assoc-D} {[M}_{A}] {[M}_{B}]-k_{diss-D} \left[ D \right]-k_{assoc-T} \left[ D \right] \left[ M_{C} \right]+k_{diss-T} [T]$$

$$\frac{d[T]}{\mathrm{dt}}=k_{\mathrm{assoc}-T} [D] {[M}_{C}]-k_{\mathrm{diss}-T} [T]$$

[concentrations]

M_a_ = 10 ?

M_b_ = 10 ?

D = 0 ?

T = 0

M_c_ = 30

Definition of the starting concentrations in µM. A question mark indicates that the starting concentration will be adjusted. In this case, adjusting the starting concentration of M_a_, M_b_ and D allows a proportion of the species to be already folded at the beginning of the reaction, which is the case at 60 °C (see thermal denaturation experiment). The starting concentration of T is assumed to be zero.

[progress]

global

file D:\T1T2T3-60-20C-dynafit\Ma.txt | response M_a_ = 1

file D:\T1T2T3-60-20C-dynafit\Mb.txt | response M_b_ = 1

file D:\T1T2T3-60-20C-dynafit\D.txt | response D = 1

file D:\T1T2T3-60-20C-dynafit\T.txt | response T = 1

[output]

directory D:\T1T2T3-60-20C-dynafit

Directories for the input and output data. The input data is a double column text file with the column being respectively, the time and the concentration of a species. The output is a html report including the rate constants and the fits.

[end]

**Supplementary References:**

[1] M. C. Jecklin, S. Schauer, C. E. Dumelin, R. Zenobi, *J. Mol. Recognit.* **2009**, *22*, 319–329.

[2] K. Akasaka, A. Naito, H. Nakatani, *J. Biomol. NMR* **1991**, *1*, 65–70.
