# Supplementary material for: Studying biomolecular folding and binding using temperature-jump mass spectrometry
Source: Nat Commun. 2020 Jan 28;11:566. doi: 10.1038/s41467-019-14179-x (PMC6987177; doi:10.1038/s41467-019-14179-x)
Supplement: Supplementary file 3 — Reporting Summary [file 41467_2019_14179_MOESM3_ESM.pdf]

nature research

Corresponding author(s): Renato Zenobi

Last updated by author(s): Dec 14, 2019

Reporting Summary

Nature Research wishes to improve the reproducibility of the work that we publish. This form provides structure for consistency and transparency in reporting. For further information on Nature Research policies, see Authors & Referees and the Editorial Policy Checklist .

Statistics

For all statistical analyses, confirm that the following items are present in the figure legend, table legend, main text, or Methods section.

n/a	Confirmed	
☐	☒	The exact sample size (*n*) for each experimental group/condition, given as a discrete number and unit of measurement
☐	☒	A statement on whether measurements were taken from distinct samples or whether the same sample was measured repeatedly
☒	☐	The statistical test(s) used AND whether they are one- or two-sided<br>*Only common tests should be described solely by name; describe more complex techniques in the Methods section.*
☒	☐	A description of all covariates tested
☐	☒	A description of any assumptions or corrections, such as tests of normality and adjustment for multiple comparisons
☐	☒	A full description of the statistical parameters including central tendency (e.g. means) or other basic estimates (e.g. regression coefficient) AND variation (e.g. standard deviation) or associated estimates of uncertainty (e.g. confidence intervals)
☒	☐	For null hypothesis testing, the test statistic (e.g. *F*, *t*, *r*) with confidence intervals, effect sizes, degrees of freedom and *P* value noted<br>*Give P values as exact values whenever suitable.*
☒	☐	For Bayesian analysis, information on the choice of priors and Markov chain Monte Carlo settings
☒	☐	For hierarchical and complex designs, identification of the appropriate level for tests and full reporting of outcomes
☒	☐	Estimates of effect sizes (e.g. Cohen's *d*, Pearson's *r*), indicating how they were calculated

Our web collection on statistics for biologists contains articles on many of the points above.

Software and code

Policy information about availability of computer code

Data collection	The data were acquired using a Waters Synapt G2S mass spectrometer. The instrument was piloted and the data recorded using MassLynx 4.
Data analysis	The data was extracted from the MassLynx files in a txt format before being treated as described in the Materials and Methods using Sigmaplot 12.5. Note that this data treatment corresponds to simple algebra and can be performed using virtually any software. In addition, for each figure, a txt file is available on the ETH server (see next section)

For manuscripts utilizing custom algorithms or software that are central to the research but not yet described in published literature, software must be made available to editors/reviewers. We strongly encourage code deposition in a community repository (e.g. GitHub). See the Nature Research guidelines for submitting code & software for further information.

Data

Policy information about availability of data

All manuscripts must include a data availability statement. This statement should provide the following information, where applicable:

- Accession codes, unique identifiers, or web links for publicly available datasets
- A list of figures that have associated raw data
- A description of any restrictions on data availability

Supplementary information is available for this paper via the publisher. The original data used in this publication are made available in a curated data archive at ETH Zurich (https://www. researchcollection.ethz.ch) under the DOIs 10.3929/ethz-b-000353280 and 10.3929/ethz-b-000385801

Field-specific reporting

Please select the one below that is the best fit for your research. If you are not sure, read the appropriate sections before making your selection.

☒ Life sciences ☐ Behavioural & social sciences ☐ Ecological, evolutionary & environmental sciences

For a reference copy of the document with all sections, see nature.com/documents/nr-reporting-summary-flat.pdf

Life sciences study design

All studies must disclose on these points even when the disclosure is negative.

Sample size	15 time points on the time-axis of the kinetics
Data exclusions	No data was excluded from the analysis, except when the equilibrium in the first block was not reached at the defined flow rate (see main text for the discussion)
Replication	The experiment were repeated as described in the main text. In particular one experiment was repeated 9 times with slightly different initial parameters. A figure is provided in the supplementary materials and is discussed in the main text.
Randomization	Not relevant.
Blinding	Not relevant. No patients involved

Reporting for specific materials, systems and methods

We require information from authors about some types of materials, experimental systems and methods used in many studies. Here, indicate whether each material, system or method listed is relevant to your study. If you are not sure if a list item applies to your research, read the appropriate section before selecting a response.

Materials & experimental systems

n/a	Involved in the study	
☒	☐	Antibodies
☒	☐	Eukaryotic cell lines
☒	☐	Palaeontology
☒	☐	Animals and other organisms
☒	☐	Human research participants
☒	☐	Clinical data

Methods

n/a	Involved in the study	
☒	☐	ChIP-seq
☒	☐	Flow cytometry
☒	☐	MRI-based neuroimaging
